# An Impulsive Suicide Attempt in a Patient with No Psychiatric History and a Recent COVID-19 Diagnosis: A case report

**DOI:** 10.1192/j.eurpsy.2022.1204

**Published:** 2022-09-01

**Authors:** J. Kim, C. Jackson, H. Raai

**Affiliations:** SBH Health System, Department Of Psychiatry And Behavioral Health, New York, United States of America

**Keywords:** Coronavirus, Covid-19, Suicide

## Abstract

**Introduction:**

The coronavirus disease 19 (COVID-19) pandemic has prompted concerns regarding increased suicide rates and exacerbation of underlying mental illness symptoms. •There is evidence suggesting neurocognitive changes as well as immune response in COVID-19 infection may increase a patient’s propensity for suicidal ideation. • Patients who are diagnosed with COVID-19 may be affected by psychological factors of anxiety, stress related to having this novel virus as well as depression, post-traumatic stress disorder and sleep disorders throughout treatment and post-treatment of continued concerns. •The combination of psychiatric, neurological, and physical symptoms associated with COVID-19 may elevate suicide risk

**Objectives:**

We present a case of a female with no prior psychiatric history who impulsively attempted suicide after a recent COVID-19 diagnosis and subsequent quarantine. Will explore possible link between increase of suicidal ideation and COVID-19 infection.

**Methods:**

A case report.

**Results:**

Link between increase of suicidal ideation and COVID-19 infection has not been clearly established but there have been reports, as in our case, of the possible vulnerability to mental illness and new onset suicidal ideation that COVID-19 survivors may experience. It may be useful to screen all patients for depressive symptoms after a COVID-19 infection. Early identification and treatment of depression in recovered COVID-19 patients will help to improve psychological impact on COVID-19 survivors and potentially reduce suicide rates.

**Conclusions:**

As COVID-19 infection may trigger new onset mental illness, exacerbate symptoms of underlying mental illness, and may increase suicidal ideation, further research is needed to evaluate links between COVID-19 infection and depression with suicidal ideation

**Disclosure:**

No significant relationships.

